# Transplanting the unexpected: a case report of a heart allograft with wild-type transthyretin cardiac amyloid

**DOI:** 10.1093/ehjcr/ytaf607

**Published:** 2025-11-25

**Authors:** Andy Jiang, Ryan Davey, Edward Tweedie, Stuart Smith

**Affiliations:** Department of Cardiac Sciences, Libin Cardiovascular Institute, The University of Calgary, 3310 Hospital Drive NW, Calgary, AB T2N 4N1, Canada; Division of Cardiology, Department of Medicine, Western University, 339 Windermere Rd, London, ON N6A 5A5, Canada; Department of Pathology and Laboratory Medicine, Western University, 1151 Richmond St, London, ON N6A 5C1, Canada; Division of Cardiology, Department of Medicine, Western University, 339 Windermere Rd, London, ON N6A 5A5, Canada

**Keywords:** Heart transplantation, Amyloidosis, Cardiomyopathy, Heart failure, Case report

## Abstract

**Background:**

Cardiac amyloidosis is increasingly recognized as a cause of heart failure. Here we present a case of a heart transplant patient that inadvertently received an allograft with wild-type transthyretin amyloidosis

**Case summary:**

A 62-year-old man with severe ischaemic biventricular dysfunction underwent an orthotopic heart transplantation. Donor heart details were limited to a report of moderate left ventricular hypertrophy, presumably from chronic hypertension. Post-operatively, the patient did well, but post-transplant imaging was suspicious for an underlying infiltrative cardiomyopathy. Endomyocardial biopsy confirmed that he had inadvertently received an allograft with wild-type transthyretin amyloidosis. Over the subsequent 7 years, he was followed in the Heart Transplant Clinic and was asymptomatic for 5 years post-transplantation. He then gradually developed signs of volume overload with serial imaging showing increased ventricular hypertrophy indicating progression of the cardiac amyloidosis. Subsequently, his condition deteriorated with multiple heart failure hospitalizations, ultimately favouring palliation, and died 7.5 years post-transplantation. Post-mortem autopsy analysis showed extensive allograft amyloid deposition without evidence of rejection or vasculopathy.

**Discussion:**

This is the first case report of a patient that inadvertently received an allograft with cardiac amyloidosis and provides insight into the natural history of this rare clinical scenario. Based on serial imaging and post-mortem histopathologic analysis, it was clear that there was progression of the underlying cardiac amyloidosis post-transplantation. We hypothesize that this occurred through the process of *amyloid seeding.* This case highlights the unique clinical and histopathologic findings of cardiac amyloidosis as well as the nuances regarding donor heart selection for transplantation.

Learning points

- To recognize cardiac amyloidosis as a cause for heart failure that has a unique clinical and histopathologic presentation
- To understand the natural history of a heart transplant patient that inadvertently received an allograft with cardiac amyloidosis

## Introduction

Cardiac amyloidosis is a disease characterized by abnormal protein aggregation and deposition in the heart interfering with normal structure and function. The two main subtypes of cardiac amyloidosis include light-chain amyloidosis (typically associated with plasma cell dyscrasias) and transthyretin amyloidosis (ATTR), which is a protein primarily produced in the liver. Transthyretin amyloidosis can be subdivided into those associated with an identifiable gene mutation (variant) and those not associated with a gene mutation (wild-type). Cardiac amyloidosis is increasingly recognized as a cause for heart failure with advances in both diagnosis and treatment.^1^ Likewise, heart transplantation has significantly improved outcomes in end-stage heart failure. While the selection of a suitable donor heart is multifaceted with definitions for ‘extended criteria’ a topic of ongoing debate,^2^ it is generally accepted that individuals with a history of known amyloidosis (with or without cardiac involvement) are disqualified from organ donation. As such, this is the first reported case of a heart transplant patient that inadvertently received an allograft with wild-type ATTR.

## Summary figure

**Table:** 

Time	Key events
Initial presentation	History of severe ischaemic cardiomyopathy, left ventricular ejection fraction < 20% Presented in acute decompensated heart failure with cardiogenic shock Stabilized with inotropic therapy and diuresis, right heart catheterization (RHC) showed acceptable parameters, and he was listed for heart transplantation
Heart transplantation	Donor details: Sixty-one-year-old Black male, neurologic cause of death Echocardiogram report notable for moderate left ventricular hypertrophy (LVH), interventricular septum thickness 16 mm, thought to be from chronic hypertension Underwent uncomplicated orthotopic heart transplantation surgery
Post-operative course	Haemodynamically stable and clinically improved Routine echocardiogram notable for significant biventricular thickening and severe diastolic dysfunction One-week post-transplant right ventricular biopsy was positive for transthyretin amyloid. No evidence of acute rejection
Year 1–3 post-transplantation	New York Heart Association Functional Class I Serial echocardiograms: preserved ejection fraction, severe LVH, diastolic dysfunction Coronary angiogram: no allograft vasculopathy
Year 4–6 post-transplantation	New York Heart Association Functional Class II Progressive volume overload requiring increasing diuretic doses Investigations: worsening LVH on echocardiograms and elevated filling pressures on RHC
Year 7–7.5 post-transplantation	New York Heart Association Functional Class IIIb–IV Multiple hospitalizations for decompensated heart failure Developed cardiorenal syndrome Overall, unfit for re-transplantation and elected for palliation
Post-mortem	Autopsy showed enlarged cardiac allograft with severe LVH Histopathology analysis showed extensive myocardial transthyretin amyloid deposition No evidence of rejection, allograft vasculopathy, or amyloid deposition in other solid organs

## Case Presentation

### Initial presentation

A 62-year-old man presented with dyspnoea, orthopnoea, and peripheral oedema. Past medical history included severe ischaemic cardiomyopathy, atrial flutter status post-catheter ablation, and a primary prevention implantable cardiac defibrillator. On examination, he was hypotensive and hypoxic and had cool extremities. Investigations demonstrated lactic acidosis, acute kidney injury, and pulmonary oedema on imaging. Overall, he was diagnosed with acute decompensated heart failure with cardiogenic shock and was started on intravenous milrinone and diuresis. Transthoracic echocardiography (TTE) demonstrated a severe biventricular dysfunction (left ventricular ejection fraction < 20%). He significantly improved with inodilator therapy, and a RHC demonstrated parameters prohibitive for a left ventricular assist device, but suitable for transplantation. After multidisciplinary discussion, he was accepted and listed for heart transplantation.

### Transplantation and post-operative course

A 61-year-old Black man was identified as a donor from a community hospital 2.5 h away following neurologic death. Pre-donation TTE (in which only the report was available) showed a preserved ejection fraction (62% by Simpson’s biplane) and moderate LVH with an interventricular septum thickness of 16 mm thought to be from documented chronic hypertension by the community hospital team. The right ventricle was normal in size with normal systolic function and mild right ventricular hypertrophy. There was no known history of carpal tunnel syndrome, peripheral neuropathy, or other conditions commonly associated with underlying amyloidosis. During organ harvest, the heart was deemed visually appropriate for transplant by the surgical team. As such, our patient underwent an uncomplicated orthotopic heart transplantation. At the time of this case, early involvement with a transplant and advanced heart failure cardiologist in donor selection decisions was not in place.

Post-operatively, the patient was haemodynamically stable and clinically well. Routine TTE showed significant biventricular thickening with severe diastolic dysfunction worrisome for potential acute rejection. Given his clinical stability, acute rejection was deemed less likely, and at this point, we had high suspicion that he had inadvertently received an allograft with an infiltrative disorder. A post-operative RHC showed elevated filling pressures, a restrictive haemodynamic pattern, normal cardiac output, and normal pulmonary vascular resistance. He continued to improve clinically, and a right ventricular biopsy performed 1-week post-transplant showed no evidence of acute rejection, but was positive for ATTR. Further molecular genetic testing confirmed wild-type ATTR on both the recipient- and donor-retained sera. This confirmed our suspicion and was disclosed to the patient.

### Follow-up and post-mortem investigations

**Figure 1:**
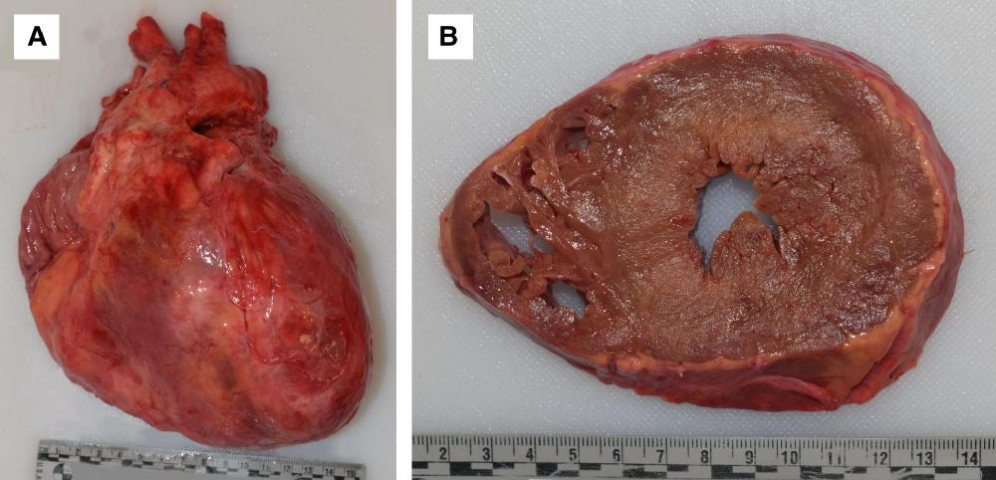
Autopsy analysis demonstrating an enlarged cardiac allograft (805 g) (*A*) and cross-sectional severe concentric left ventricular hypertrophy (*B*).

**Figure 2:**
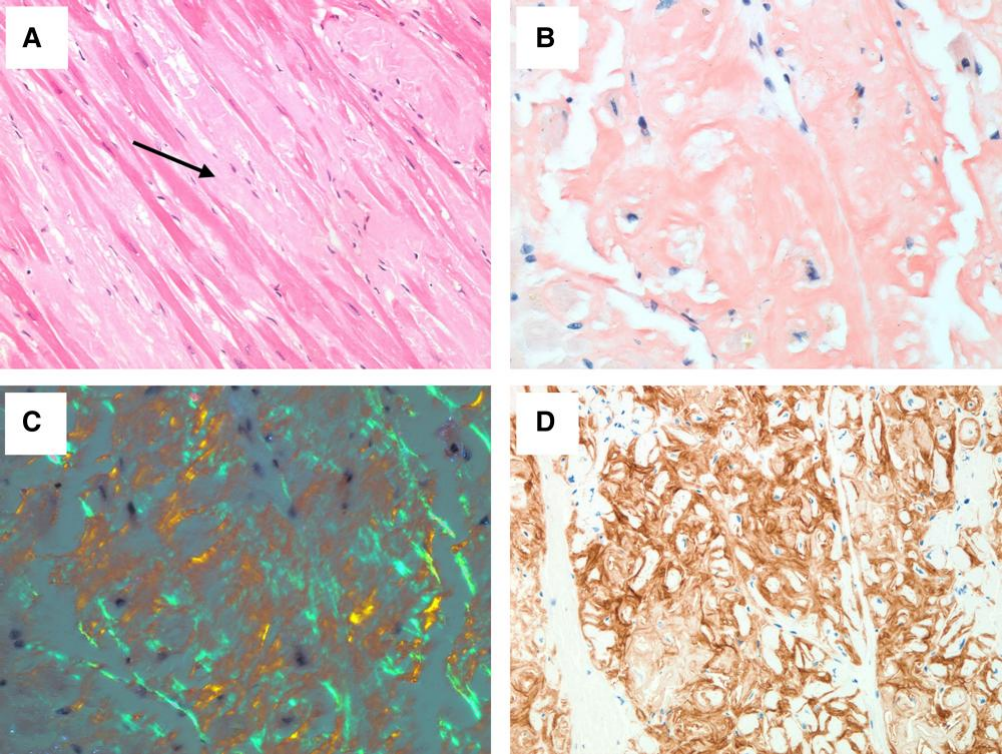
Post-mortem histopathology of the cardiac allograft demonstrating extensive transthyretin amyloid deposition: haematoxylin and eosin stain (*A*) with pink interstitial amyloid (arrow). Positive Congo Red stain (*B*) with classic ‘apple-green’ birefringence with polarized light (*C*). Amyloid protein demonstrating strong immunohistochemical brown chromogen staining for transthyretin (*D*).

Post-transplant, the patient was on triple immunosuppression with tacrolimus, mycophenolate, and weaning of steroids as per local protocol. There was no biopsy evidence of rejection. From a functional standpoint, he achieved New York Heart Association (NYHA) Class I. He had no evidence of systemic hypertension. There was no allograft vasculopathy on routine left heart catheterization. Serial TTE post-transplantation demonstrated progressive severe LVH, elevations in estimated pulmonary pressures, and a reduction in right ventricular systolic function (*Videos 1 and 2*). Likewise, serial RHC demonstrated worsening filling pressures in a restrictive haemodynamic pattern (*Table 1*). He remained stable at NYHA Class I until approximately Year 5 post-transplant when he progressed to NYHA Class II symptoms and then began to require escalating diuretic doses. In his seventh-year post-transplant, he further declined with NYHA Class IIIb–IV symptoms, repeated heart failure hospitalizations, deconditioning with cachexia, and development of cardiorenal syndrome and was ultimately deemed not suitable for re-transplantation. He was palliated and died 7.5 years post-transplantation. An autopsy was performed, which demonstrated an enlarged cardiac allograft weighing 805 g with severe concentric LVH (*Figure 1*). Histopathology demonstrated extensive myocardial ATTR deposition (*Figure 2*). There was no evidence of rejection, allograft vasculopathy, or amyloid deposition in other solid organs.

**Table 1:** Temporal trends in right heart catheterization data and key echocardiographic parameters

Measured parameter	Pre-transplant	3 months post-transplant	1 year post-transplant	3 years post-transplant	5 years post-transplant
RA	22	3	8	20	18
RV	79/20	43/2	50/8	64/20	56/18
PA	83/37 (53)	43/18 (27)	50/23 (35)	58/23 (38)	58/21 (38)
PCWP	33	21	22	24	22
CI, Fick method	1.1	2.0	2.2	1.8	1.7
PVR	9	2	3	4	4
Square root sign	Absent	Absent	Absent	Present	Present
Equalized diastolic pressures	Absent	Absent	Present	Present	Present

RA, mean right atrial pressure, mmHg; RV, right ventricular systolic and diastolic pressure, mmHg; PA, pulmonary artery systolic, diastolic, and mean pressure, mmHg; PCWP, mean pulmonary capillary wedge pressure, mmHg; CI, cardiac index by Fick method, L/min/m^2^; PVR, pulmonary vascular resistance, Wood units.

## Discussion

Though rare, there have been previous reports of patients with no prior history of amyloid then developing wild-type cardiac amyloidosis over a decade after heart transplantation.^3,4^ To our knowledge, this is the first reported case of a patient that inadvertently received a cardiac allograft with wild-type ATTR. From review of serial TTEs with increasing LVH, it was clear that the cardiac amyloidosis progressed independent of any pre-existing or new amyloid elsewhere in the body, nor did he have hypertension, aortic stenosis, or allograft rejection to explain worsening LVH. While calcineurin inhibition with tacrolimus has been previously associated with developing cardiac hypertrophy,^5,6^ the more likely explanation in this case is progression of the amyloidosis as seen in histopathology analyses.

Though the mechanism for how the amyloid progressed is not clear, a potential hypothesis may be found in the idea of ‘amyloid seeding’. This previously described theory postulates that the kinetics of pathologic amyloid protein aggregation and deposition into organs rapidly increased in the presence of pre-existing ‘seeds’ of amyloid.^7^ As such, for this case, the pre-existing seeds of amyloid in the allograft heart potentially allowed for rapid pathologic deposition of amyloid produced by the native liver. A potential treatment option could include transthyretin protein stabilizers (tafamidis or acoramidis), or a transthyretin gene silencer (vutrisiran), that have been shown to reduce morbidity and mortality in variant or wild-type ATTR cardiomyopathy.^8–11^ However, their role in heart transplant patients is unclear. None of these medications were routinely available at the time for this patient. Lastly, there is no defined role for orthotopic liver transplantation for the treatment of wild-type ATTR.

The question also remains as to how this case could have been prevented. From a systems perspective, ensuring access to not only TTE reports but also images is vital as those with expertise in heart failure might have noticed signs of an underlying infiltrative cardiomyopathy. There are many TTE findings that, when present, would raise suspicion for underlying amyloidosis.^12^ However, timely access to advanced imaging modalities and results may not always be possible depending on local availability and expertise. Early involvement of a multidisciplinary team including advanced heart failure/transplant cardiologists along with cardiac surgeons is therefore essential in making important decisions regarding donor suitability and identifying potential red flags. As a result of this case, protocols were set in place at our institution such that donor heart decisions would always involve a multidisciplinary team that included a transplant cardiologist.

The initial reported interventricular septum thickness of 16 mm from presumed chronic hypertension should also raise concern, as septal thickness in hypertensive heart disease is typically <15 mm.^13^ However, broadly speaking, LVH by itself is not an absolute contraindication to organ donation by recent international guidelines.^2^ In fact, prior research suggests that outcomes using allografts with even severe LVH are favourable, especially in younger donors and low ischaemic time scenarios.^14,15^ Most recent international guidelines provide no specific recommendations regarding donor investigations for LVH, or screening for the presence of cardiac amyloidosis in donor hearts.^2^ Rather, a holistic review of the recipient, donor, investigations, and a degree of clinical suspicion should inform clinical decisions regarding eligibility.

## Conclusion

Cardiac amyloidosis is increasingly recognized as a cause for heart failure with a unique clinical presentation and histopathologic presentation. While unfortunate, this case has provided valuable insight into the natural history of this rare clinical scenario and reinforces the importance of multidisciplinary clinical decision-making with respect to donor organ selection.

## Lead author biography


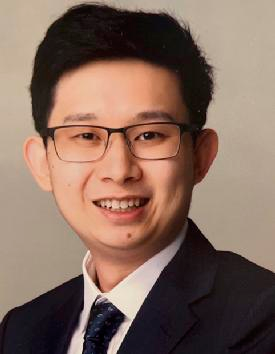


Dr Andy Jiang completed his medical school at the University of British Columbia, Vancouver, BC, Canada, and subsequently completed his residency in Internal Medicine at the University of Western Ontario, London, ON, Canada. He is currently a cardiology fellow at the Libin Cardiovascular Institute at the University of Calgary, AB, Canada with a broad interest in various areas of cardiology.

## Data Availability

The data underlying this article are available on reasonable request to the corresponding author.

## Notes

**Consent:** The authors confirm that informed consent for this publication was provided by the next of kin in accordance with COPE best practice guidelines.
